# 

**Published:** 1921-11

**Authors:** 


### OUR LEAFLET COMPETITION.

### THE CARE OF THE TEETH.

Some of our competitors had not taken the trouble to read our Rules, and sent us, in consequence, leaflets that far exceeded the three-hundred-word limit. Of the excellence of these lengthy effusions we cannot judge, for we didn't read them. Nor did we read the leaflets that began with "Caries causes countless calamities," and "Dental decay deals death to dozens daily." Several leaflets appeared to have a vague moral and religious undercurrent; and one, obviously inspired by one of the worst efforts in Hymns Ancient and Modern, began:—

"When morning wakes the thrush
I take a little brush
And clean my dirty teeth. . . ."

Several leaflets were anecdotal, and ended with an underlined moral after the manner of *The Fairchild Family*; and one, apparently, was a piece of autobiography, and referred to the difficulty of enjoying a denture and toffee simultaneously.

Leaflets submitted by *Namder, Toothpick, Union, Bon Accord, Anne Oviss*, and *Odons* were of merit. An illustrated leaflet was sent to us by *The Children's Friend*, of which the pictures were excellent; but we had not asked for pictures. Several other competitors, notably *Dens, Fluffy*, and *Regina Virida* sent us good leaflets.

We divide our prize of three guineas equally among *Uncle Salus, Teddy*, and "*One who wears his Own Teeth at 36*." The first two prize-winners succeeded in writing good and almost monosyllabic English, the third prepared for us a lovely poster-leaflet.

### THE PRIZE LEAFLETS.

### ( 1 )

**The Care of the Teeth.**

Very many people are ill because they have bad teeth.

If you were to suck a piece of bad meat or a dead mouse you would soon be ill, and if you have dead and rotten teeth in your mouth it is just as bad, for you are swallowing poison from them all day long and every night. The poison from these rotten teeth will make you ill, and a sick person is always unhappy, and often cannot earn his own living.

It is much better to be well than ill, and to be happy instead of miserable; and the best way to be well and happy is to have good teeth.

If you clean your teeth every night and every morning they will not become bad; they only go rotten because they are not cleaned. So brush your teeth every night before going to bed, and do not eat any sweets after you have cleaned them; then brush your teeth first thing in the morning.

Apples and crusts and food that take a lot of chewing are very good for the teeth; so the more you can eat of these the better. If you ever get toothache always go and see a dentist at once, or if you know that one of your teeth is bad, then go and see a dentist about it.

It is very important not to keep one bad tooth in your mouth, even if it is a first tooth. One bad apple in a cask may make all the others turn bad, and one bad tooth in your mouth may make all the others go rotten.

*Teddy.*

### ( 2 )

**The Care of the Teeth.**

*Why use a Tooth-brush?*

BECAUSE a tooth-brush is of little use without a tooth-paste, and some trade-preparations are expensive, many MOTHERS, who did not use the tooth-brush in their own school-days, regard the tooth-brush as a FAD.

THESE MOTHERS ARE NOW TOOTHLESS, unless the dentist has re-furnished their gums.

BUT

SUCH MOTHERS scrub their frying-pans after use, SCOUR their sauce-pans, and carefully CLEAN their spoons, forks and pastry-boards. YET to CLEAN pots and pans before preparing the FAMILY'S MEALS is USELESS IF THE MOUTH IS NOT EQUALLY CLEAN into which the cooked food goes. What the PAN is to the cook the MOUTH is to the EATER; and to PUT CLEAN FOOD INTO A DIRTY MOUTH is as FOOLISH as to keep fresh MILK

IN A SLOP PAIL.

A DIRTY MOUTH soils food, breeds germs, and discharges them into the STOMACH.

To stuff your STOMACH with GERMS is WORSE than to go supperless.

THE CHILD who uses a TOOTH-BRUSH is the child whom the DENTIST most quickly SENDS AWAY.

To KEEP your TEETH employ your TOOTH-BRUSH. To SAVE yourself from TOOTHACHE BRUSH YOUR TEETH.

TEETH should be BRUSHED EVERY NIGHT before bed-time, and, preferably, also every morning before breakfast.

DO YOU WANT

FOUL BREATH, DECAYED TEETH, DISCHARGING GUMS, INDIGESTION, RHEUMATISM, and LARGE DOCTORS' BILLS?

IF SO

YOU can HAVE THEM ALL by "Saving the price" of a TOOTH-BRUSH.

BUT IF NOT

Buy a Tooth-brush and use it

NIGHTLY FOR 3 MONTHS.

NO BRUSH WILL LAST CLEAN for longer, but if you replace the brush, YOUR HEALTH WILL.

*By One who Wears his Own Teeth at 36.*

**The Prize Leaflets - (*cont.*).**

( 3 )

**The Care of the Teeth.**

Would you like to know how to prevent toothache? You would? Then you must first learn how to kill germs. It is quite easy, even though they are too small to see. You are perhaps surprised to hear that you have germs in your mouth. They are there, nevertheless, and love to feed on the bits of food and sugary liquids that stick to your teeth after meals or after eating sweets.

If you do not kill these germs, poisons are formed. Some of these poisons will destroy your teeth and give you toothache, and some of them you will swallow—especially while you are asleep. The whole body will thus become poisoned.

If you brush your teeth properly before going to bed (and keep to the rules below), the germs will have nothing to feed on and all will be well.

These are the rules of the game:—

Rules for the Care of the Teeth.

1. Clean the teeth last thing *every* night and first thing *every* morning.
2. Do not eat or drink anything, except water, after cleaning the teeth at night.
3. In cleaning the teeth, work the brush up and down on all sides, getting into the crevices of both the double and single teeth. Camphorated chalk, carbolic tooth-powder, any good tooth-paste, or even soap, are all good to use.
4. Eat plenty of hard or tooth-cleansing foods, such as crusty bread, apples, etc., as often as you can.
5. After eating sweets, rinse the mouth out with plenty of water.

Follow these simple rules *every* day.

P.S.—If, in spite of all your care, you should get toothache, go and see a dentist.

*Uncle Salus.*

### A Leaflet Competition

***We offer Three Guineas for the best new and original leaflet of not more than 300 words on***

**"CLEANLINESS"**

**The Leaflet must be suitable for circulation in the Public Elementary Schools and among the Scholars of the Secondary Schools. The result of this Competition will be announced and the successful Leaflet will be printed in the December Number of "The Hospital and Health Review." We reserve the right to republish any of the Leaflets submitted to us.**

**RULES AND REGULATIONS.**

1. This Competition is open to all.
2. Leaflets must be written or typed on unprinted paper on one side of the page only.
3. Competitors must employ a pseudonym.
4. Leaflets must be received by The Editor at "The Hospital and Health Review," 28 Southampton Street, Strand, W.C. 2, by **the first post on November 26, 1921.** Envelopes must be marked in the top left-hand corner with the word "Leaflet."
5. The Editor's decision is final.

### BOOKS RECEIVED.

W. Heinemann, Ltd.: "Diathermy: its Production and Uses in Medicine and Surgery." By E. P. Cumberbatch, M.A., B.M., M.R.C.P. 21s. net. "A Textbook of General Pathology." By J. M. Beattie, M.A., M.D., M.R.C.S., L.R.C.P., and W. E. C. Dickson, M.D., B.Sc., F.R.C.P. 31s. 6d. net.

J. & A. Churchill: "Notes on Diseases Treated by Medical Gymnastics and Massage." By Dr. J. Arvedson. Translated and edited by Mina L. Dobbie, M.D., B.Ch. 8s. 6d. net.

### APPOINTMENTS.

### Institutional.

Miss Amy F. M. Horne to be matron to Ripley Cottage Hospital.

Miss Dorothy Jones to be matron to St. Chad's Hospital, Birmingham.

Miss H. Powell Evans to be matron to Dean's Isolation Hospital, South Shields.

Miss Isabel Mary Middleton to be matron to the Hospital for Sick Children, Newcastle-on-Tyne.

Mr. John Kirk, M.A., B.Sc., M.B., Ch.B.Glas., D.O.Oxon., to be Honorary Assistant Ophthalmic Surgeon to the Royal Eye and Ear Hospital, Bradford.

### Public Health.

Mr. W. E. Thomas, L.R.C.P.&S.Edin., to be Medical Officer of Health for Bridgend, Glam.

Mr. J. O. Ward, M.R.C.S., L.S.A., to be certifying surgeon under the Factory and Workshops Act for Knottingley.

Mr. J. M. Milne, L.R.C.P.&S.Edin., Mr. W. A. Reynolds, M.B., Ch.B., and Mr. J. McTurk, L.R.C.P.&S.Edin., to be certifying surgeons under the Factory and Workshops Acts to Carnoustie, Paignton, and Newton Stewart respectively.

Miss Mona Carr (trained at the Borough Hospital, Birkenhead) to be health visitor under the Battersea Borough Council.

Miss E. L. Griffiths (trained at the Royal Southern Hospital, Liverpool) to be health visitor to the Herefordshire County Council.

Miss A. B. Pilkington (trained at Guy's Hospital) to be health visitor to the Folkestone (Urban District) Borough Council.

Miss Rosa Cross (trained Hunslet Infirmary) and Miss K. M. Banks (trained Fir Vale Hospital, Sheffield) to be health visitors for the county borough of Derby.

Miss Elizabeth J. Haighton (trained at St. Bartholomew's Hospital), Miss Ethel M. Slaney (trained at the Radcliffe Infirmary, Oxford), Miss Annie Watts (trained at Manchester Royal Infirmary), and Miss Florence E. Weaver (trained at Southport Infirmary) to be health visitors to the Corporation of the City of Manchester.

### RECENT OFFICIAL PUBLICATIONS.

[To be obtained at H.M. Stationery Office, Imperial House, Kingsway, W.C. 2. The prices include postage.]

Education Syllabus of Physical Training for Colleges. Two Years' Course. 4d.

Stationery Office. Special Report, Series No. 61. Experimental Rickets (Medical Research Council). 4s. 3d.

Parliamentary Annual Report of the Chief Medical Officer of the Board of Education, 1920. Cmd. 1,522. 6s. 3½d.

Report on Public Health and Medical Subjects, No. 6. Incidence of Notifiable Infectious Diseases in each Sanitary District in England and Wales during the year 1920. 2s. 1½d.

Statutory Rules and Orders, 1921. No. 1,594. National Health Insurance (Insurance Committees) Amendment Regulations (Bo. 4), October 17, 1921, with regard to the Constitution of Insurance Committees in Wales. 2d.

### MANAGERIAL NOTICES.

**All letters on business matters must be addressed to The Manager, "The Hospital and Health Review," 28 and 29 Southampton Street, London, W.C. 2.**

RATES FOR SUBSCRIPTION (payable in advance).

| | | | |
|---|---|---|---|
| Three Months (including Postage) | | ... ... | 2s. 0d. |
| Six Months | do. | ... ... | 3s. 9d. |
| Twelve Months | do. | ... ... | 7s. 6d. |

Subscriptions (which may commence at any time) are payable in advance. Cheques and Post-Office Orders (crossed London County and Westminster Bank, Covent Garden Branch) should be made payable to the Manager of THE HOSPITAL AND HEALTH REVIEW.

**It is especially requested that remittances be made by Cheque or Postal Order and *NOT* Stamps.**

***Advertisements.*** Scale of charges and particulars of special positions may be obtained on application to the Manager.

